# A Systematic Review of Scientific Studies on the Effects of Music in People with Personality Disorders

**DOI:** 10.1192/j.eurpsy.2023.425

**Published:** 2023-07-19

**Authors:** R. Haslam, A. Heiderscheit, H. Himmerich

**Affiliations:** ^1^Mental Health Studies Programme, King’s College London, London, United Kingdom; ^2^Department of Music Therapy, Augsburg University, Minneapolis, United States; ^3^Department of Psychological Medicine, King’s College London, London, United Kingdom

## Abstract

**Introduction:**

Personality Disorders (PDs) are psychiatric conditions involving maladaptive personality traits and behaviours. Previous research has shown that musical preferences and the use of music may be related to personality traits. Additionally, music therapy (MT) is increasingly being used as a treatment option for people with PDs.

**Objectives:**

This systematic review aimed to summarise the findings of the existing literature on music, MT, and PDs, and to identify any gaps in the existing literature.

**Methods:**

Using the PRISMA guidelines, a systematic literature search was undertaken using three databases: PubMed, Web of Science, and PsycInfo.

**Results:**

A total of 24 studies were included in this review and summarised into four categories: music preference, MT, music performance, and music imagery, all in relation to PDs or traits associated with PDs. The analysis found that individuals with personality traits associated with PDs may prefer different types or genres of music or interact with music differently than those without these traits. Additionally, MT was found to offer a potentially useful treatment option for PDs.

**Conclusions:**

The power of these findings was limited by the small number of included studies. This review offers a useful foundation upon which further research looking at MT as a potential treatment option for PDs can be built. As selected music has been reported to help to reduce violence and hostility, patients may develop playlists with the support of their therapists to manage aggression and violent impulses in foreseeably difficult situations; appropriate music for bedtime relaxation can be recommended to improve sleep length and quality; and people who experience insecurities may be encouraged to try music to aid cognitive problem solving and improve their mood.

**Disclosure of Interest:**

None Declared

